# Predictive models for occurrence of expansive intracranial hematomas and surgical evacuation outcomes in traumatic brain injury patients in Uganda: A prospective cohort study

**DOI:** 10.21203/rs.3.rs-3626631/v1

**Published:** 2023-11-25

**Authors:** Larrey Kasereka Kamabu, Ronald Oboth, Godfrey Bbosa, Ssenyondwa John Baptist, Martin N. Kaddumukasa, Daniel Deng, Hervé Monka Lekuya, Louange Maha Kataka, Joel Kiryabwire, Galukande Moses, Martha Sajatovic, Mark Kaddumukasa, Anthony T. Fuller

**Affiliations:** Makerere University; Makerere University; Makerere University College of Health Sciences; Makerere University; Makerere University; Duke University; Makerere University; Faculty of Medicine, Université Catholique du Graben; Makerere University; Makerere University; University Hospitals Cleveland Medical Center & Case Western Reserve University School of Medicine; Makerere University; Duke University

**Keywords:** Traumatic expansive intracranial hematomas, predictive models, occurrence, and neurosurgical outcomes

## Abstract

**Background:**

Hematoma expansion is a common manifestation of acute intracranial hemorrhage (ICH) which is associated with poor outcomes and functional status.

**Objective:**

We determined the prevalence of expansive intracranial hematomas (EIH) and assessed the predictive model for EIH occurrence and surgical evacuation outcomes in patients with traumatic brain injury (TBI) in Uganda.

**Methods:**

We recruited adult patients with TBI with intracranial hematomas in a prospective cohort study. Data analysis using logistic regression to identify relevant risk factors, assess the interactions between variables, and developing a predictive model for EIH occurrence and surgical evacuation outcomes in TBI patients was performed. The predictive accuracies of these algorithms were compared using the area under the receiver operating characteristic curve (AUC). A p-values of < 0.05 at a 95% Confidence interval (CI) was considered significant.

**Results:**

A total of 324 study participants with intracranial hemorrhage were followed up for 6 months after surgery. About 59.3% (192/324) had expansive intracranial hemorrhage. The study participants with expansive intracranial hemorrhage had poor quality of life at both 3 and 6-months with p < 0.010 respectively. Among the 5 machine learning algorithms, the random forest performed the best in predicting EIH in both the training cohort (AUC = 0.833) and the validation cohort (AUC = 0.734). The top five features in the random forest algorithm-based model were subdural hematoma, diffuse axonal injury, systolic and diastolic blood pressure, association between depressed fracture and subdural hematoma. Other models demonstrated good discrimination with AUC for intraoperative complication (0.675) and poor discrimination for mortality (0.366) after neurosurgical evacuation in TBI patients.

**Conclusion:**

Expansive intracranial hemorrhage is common among patients with traumatic brain injury in Uganda. Early identification of patients with subdural hematoma, diffuse axonal injury, systolic and diastolic blood pressure, association between depressed fracture and subdural hematoma, were crucial in predicting EIH and intraoperative complications.

## Introduction

Traumatic brain injury (TBI) is common around the world, burdening economies with substantial healthcare costs in treatment and long-term neuropsychological disability ^1^. Globally, TBI affects at least 69 million individuals and accounts for 5 million deaths annually^2^. Of these, 93% occur in low- and middle-income countries (LMICs) ^3–5^. The highest cases of TBI result from road traffic accidents (RTA) ^4^. In Uganda, mortality among TBI patients is 4.2 times higher than in the US^6,7^. TBI is a major cause of prolonged sequelae, death in the young population, and a social burden as well ^8–11^. TBI encompasses numerous types of insults to the brain, including scalp injury, skull fractures, surface contusions, diffuse axonal injury (DAI), hypoxic-ischemic damage, meningitis, and intracranial hemorrhage ^12^. Among the most serious consequences is expansive intracranial hematoma (EIH). EIH refers to evidence of increased hematoma volume of over 33% within the intracranial vault or absolute hematoma growth over 6mL from an initial scan with varying consequences ^13^. Intracranial hemorrhages can be classified in three ways: (1) based on time, classified as hyperacute, acute, subacute, and chronic; (2) based on size, including small, big, and massive; and (3) based on location, classified as epidural, subdural, intracerebral, intraventricular and subarachnoid ^14^.

Currently, there is no effective treatment strategy to prevent EIH development in patients with TBI ^15–19^. There is no clear effective therapy for EIH, thus also affecting the time of interventions making them experience varying prognoses, which at times can be poor ^20,21^. Although there have been advances in quick and accurate diagnostic imaging procedures, the related morbidity and mortality following EIH are persistently high ^13^. EIH is a critical predictor of poor prognosis in TBI patients, with varying incidences of progression of hematoma between 13 to 38% ^22^. It is necessary to assess the risk of EIH in patients with intracranial hematoma (IH) as early as possible during hospitalization to avoid unnecessary morbidity and poor prognosis. The subdural hematoma (SDH), skull fracture, subarachnoid hemorrage (SAH) have been identified as risk factors for EIH among IH patients using logistic regression ^12,23–26^.

Some studies propose different prognostic models, for example, the ICH score, to assess which patients would benefit from surgical treatment and which from a conservative one. A problem they present is that they do not take into account the evolutionary nature of intracranial hematomas^14^.

The prospect of modeling has gained attention and excitement because of its advantages in processing large and high-dimensional medical data. It might offer a practical tool to enhance resource allocation in remote settings with limited resources. Accurate predictive models for EIH occurrence and unfavorable surgical evacuation outcomes could be derived from early biological markers of injury severity. Currently, no studies have investigated the accuracy of modeling algorithms in predicting EIH and surgical evacuation outcomes in patients with IH in Uganda. Therefore, this study was conducted to identify relevant risk factors, assess the interactions between variables, and establish a predictive model for EIH occurrence and neurosurgical outcomes in TBI patients at MNRH.

## Material and methods

### Study design

A prospective cohort study conducted between the June 2021 and June 2023 to predict EIH occurrence and surgical evacuation outcomes in TBI patients.

### Study settings

The study was conducted at Mulago National Referral Hospital (MNRH), Kampala, Uganda. MNRH, the largest public hospital in Uganda located around 5 kilometers from the city center. It serves about 75% of injured victims in Kampala and surrounding districts ^27^. The Accident and Emergency department attends to approximately 64 urgent surgical evacuations of intracerebral hemorrhage in patients with acute TBI at MNRH every month ^28^. TBI patients were recruited on admission at the Accident and Emergency Department, then followed up in the operative theatres and the neurosurgery department.

### Study population

All TBI patients aged 18 years or older with a radiologically confirmed brain computerized tomography scan that showed an intracranial bleed were eligible to participate in the study. Purposive sampling was used to recruit participants.

### Study participant selection criteria

Participants were TBI patients aged 18 years and above, post resuscitation GCS of 4 to14, with evidence of EIH on two CT scans (increase in hematoma volume > 33% or absolute hematoma growth > 6ml from the initial scan) exclusively, eligible for cranial surgery and enrolled in the study within 24 hours of initial presentation to hospital. A written signed informed consent from the patient or their next of kin was obtained. Patients with (1) known pre-thrombocytopenia, (2) a history of coagulation disorders, and (3) used anticoagulants, (4) pregnancy, (5) and those with the inability to consent before surgical intervention were excluded from the study.

EIH was defined by an increase in acute intracranial traumatic hematoma volume > 33% or absolute hematoma growth > 6ml from the initial scan within 72 hours of injury during the study period^29^

### Study procedures and variables

Patients were recruited from their admission at the Accident and Emergency Department, followed up in the operative theatres, postoperatively in the neurosurgery ward, and neurosurgical outpatient clinics for up to 6 months for the occurrence of complications. Trained research assistants, using a Research Electronic Data Capture (REDCap) system, collected pertinent demographic, clinical, laboratory, and radiological information. Patient demographic characteristics recorded included age, gender, occupation, residence type, geographic regions, and matrimonial state. Outcome’s data were obtained at enrollment (baseline) and then at 1 day, 30-, 90-, and 180-days post-discharge. Clinical outcomes were recorded during these outpatient visits. The principal outcomes included death from any cause and complications (clinically apparent spasticity, cerebrospinal fluid leakage, bleeding diastasis, infection, and pneumonia). Other parameters of concern included type of cranial surgery (burr holes, craniotomy, craniectomy), evolution of intracranial hematomas over time (within 24, > 24 but < 48, and over 72 hours), timing to surgery (early, late and delayed), and site of surgery (frontal, fronto-parietal, fronto-parieto-temporal, parieto-temporal, occipito-parietal, temporal, parietal, occipital, interhemispheric, or posterior fossa). Early surgical evacuation occurs when an expansive mass lesion is removed early (within 24 hours) after an acute TBI^30^. Late surgical evacuation occurs when the expansive mass lesion is removed later (within 25 to 72 hours) after an acute TBI ^30,31^. Delayed surgical evacuation occurs when an expansive mass lesion is removed 72 hours after an acute TBI.

### Statistical data analysis

Non-parametric continuous data were summarized using the median and interquartile range, whereas continuous parametric data were shown using the mean and standard deviation. Categorical variables were expressed as frequencies and percentages. To assess for the risk factors of expansive hematoma, the chi-square test was used at bivariate to assess for variables that had an independent association with the outcome.

We used logistic regression model and selected variables and those of clinical significance by a stepwise backwards selection based on model AICs. The explanatory variables were categorized into demographical features, neurological assessment, baseline laboratory characteristics, baseline blood pressure characteristics, and neuroimaging patterns. Only the first orders of the variables were included at this step. Then, implemented the model selection again by including all the interaction terms of the selected variables in the previous step. The model selection procedure is the same and based on model AICs. Eventually, we pooled all the selected variables together and manually picked the most relevant variables. All the variables and their interaction terms were included in this model which was later under the same screening process as previous models. The study evaluated the model performance using AUC, accuracy, and five-fold cross-validation.

The study adapted this metric-based and manual mixed screening procedure, because of the large number of candidate variables collected in the data. The metric-based screening was to eliminate statistically irrelevant factors from the data, while the manual screening was based on neurological knowledge and the purpose of having an interpretable model. It was likely that some variables were missed, but we believe that this procedure was both feasible and suitable.

The data were analyzed using regression models that fit the response type: logistic for binary, ordinal logistic for ordinal variable, and linear for continuous. Variables with p-values < 0.05 remained in the model and were considered to be significant risk factors for intra and post-operative complications, including death among adult patients with expansive hematomas following traumatic brain injury. Then, variables that “best” fit the outcome (i.e., EIH) were selected by choosing the model with the lowest AIC during a backward stepwise selection. Finally, all selected variables were pooled from each category to fit the final logistic regression model. The final model was selected based on the same criteria, but the predictive performance on the validation dataset was undesirable. Therefore, the model was manually tweaked to achieve better accuracy, AUC in both validation dataset and five-fold cross-validation. Levels of p < 0.05 were considered statistically significant.

### Study Flow of the Participants

**Figure 1:**
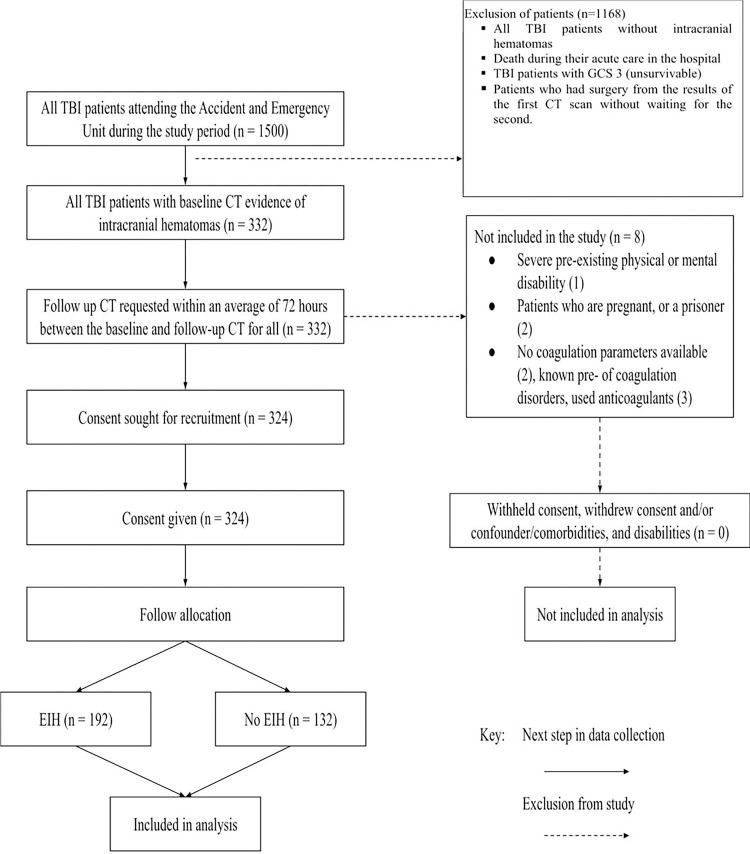
Recruitment flow chart

During the study period, a total of 1500 patients were screened (Fig. 1). Out of these, 21.6% (n = 324) were enrolled into the study. In a cohort of 324 patients with intracranial hematomas, 59.3% (n = 192) had expansive intracranial hematomas indentified on CT scan (Fig. 1). Of the 324 patients with intracranial hematomas, 192 (59.3%) had expansive hematomas identified on CT scan resulting in a proportion of 0.59 (95% CI: 0.54 to 0.65).

### Demographic characteristics

In this study, a majority of the participants with EIH were significantly older than the no EIH group (42.3 ± 17.9 vs. 30.5 ± 14.0), most of the patients were male 261 (80.6%) and majority 152 (46.9%) were motorcyclists known as boda riders. The results show that most of the patients 184 (56.8%) were from rural residence (Table 1).

### Proportion of traumatic brain injury patients presenting with expansive hematomas

Of the 324 patients with intracranial hematomas, 59.3% (n = 192) had expansive hematomas identified on CT scan.

### Models leading to expansive intracranial hematoma development following TBI

In the final model, age, systolic blood pressure, diastolic blood pressure, subdural hematoma (SDH), diffuse axonal injury (DAI), skull fracture, and an interactive term of skull fracture and SDH were selected. Odds of having EIH increased to 1.045 times for one increment in systolic blood pressure. Odds decreased to 0.942 times for one increment in diastolic blood pressure. Having SDH increased the odds of EIH to 6.286 times, while diffuse axonal injury increased it to 4.024 times. Given a patient has skull fracture, SDH decreased the odds of EIH to 0.0676 times (Table 2).

**Figure 2:**
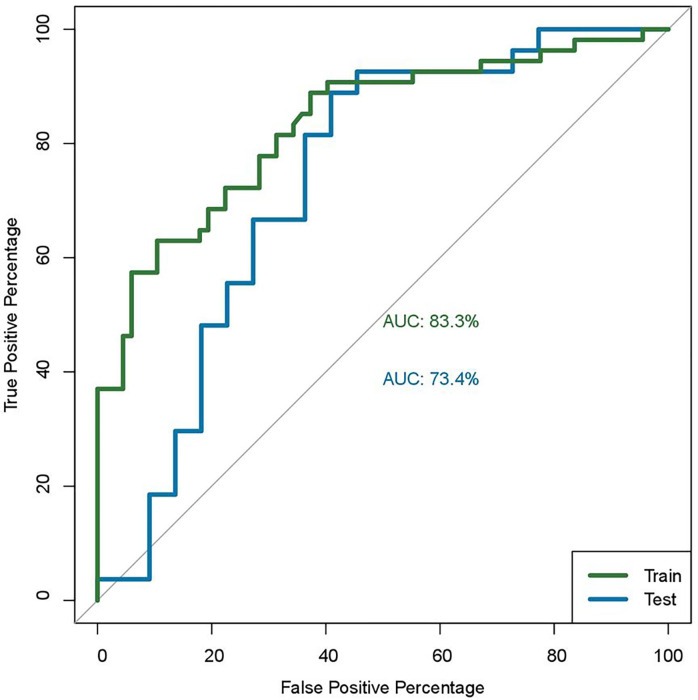
ROC curve figure for predictive model for EIH occurrrence with two curves, one for training data, one for testing data.

Figure 2 has two curves, one for training data, one for testing data. Model demonstrated good discrimination with areas under curve for EIH occurrence (0.833) while the average accuracy was 73.4%.

### Postoperative clinical Outcomes

The respective inpatient postoperative risks were 10.2% for death, 58.0% for intraoperative complications, 15.7% for early posttraumatic seizures (PTS), 11.7% for coma, 11.7% for brain oedema, 4.9% for wound infection, 4% for nutrition deficit, 3.4% for pneumonia, 2.8% for CSF leakage, 1.2% for subdural hygroma (Table 3). However, 282(88.4) alive until the end of the study. Among these patients, 208 (73.8%) were discharged with favourable condition (defined by GOS of > 3), 74 (26.2%) were discharged with unfavourable condition (defined by GOS of < 3).

### 18-month Survival of TBI patients with expansive intracranial hematomas after neurosurgical evacuation

**Figure 3:**
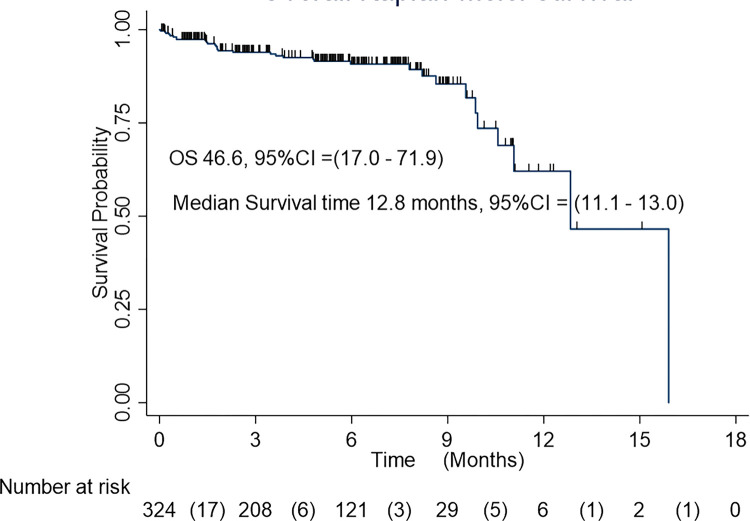
Surgical outcomes of EIH among TBI patients

In this study, 324 TBI patients with intracranial hematomas after surgery were followed up for a total of 15.9 months and the overall survival was 46.6%, 95% CI= (17.0 to 71.9) based on Kaplan–Meier curve. The median survival time was 12.8 months, 95% CI= (11.1 to 13.0). The estimated cumulative survival was 97.38% (95% CI: 94.83–98.68) within the first month of follow-up, 94.35% (95% CI: 90.92–96.50) after 2 months of follow-up, 93.95% (95% CI:90.43–96.20) after 3months of follow-up,90.81% (95% CI: 86.32–93.87%) after 6 months of follow-up, 62.07% (95% CI: 40.35–77.83) after 12 months of follow-up, and 46.55 (95% CI: 16.96–71.93) after 15.9 months of follow-up. The probability of survival decreased as the follow-up time increased, especially within the first months of follow-up according to the Kaplan–Meier survival curve for time to death for TBI patients with intracranial hematomas after surgery (Fig. 3).

### Models that can predict intraoperative complications after neurosurgical evacuation in traumatic expansive intracranial hematoma patients

Holding the rest of variables constant, undergoing craniectomy would make the odds of getting intraoperative complication become 8.5317969 times (p = 0.03578). Holding the rest of variables constant, undergoing craniotomy would make the odds of getting intraoperative complications become 1.5244901 times (p = 0.56229). Holding the rest of variables constant, having delay surgery (over 72 hours from the accident) would make the odds of getting intraoperative complications become 2.8757974 times (p = 0.00229). Holding the rest of variables constant, having early surgery (within 24 from the accident) would make the odds of getting intraoperative complications become 1.6229086 times (p = 0.27600). Holding the rest of variables constant, undergoing frontoparietal decompression would make the odds of getting intraoperative complications become 3.6784446 times (p = 0.00531).

**Figure 4:**
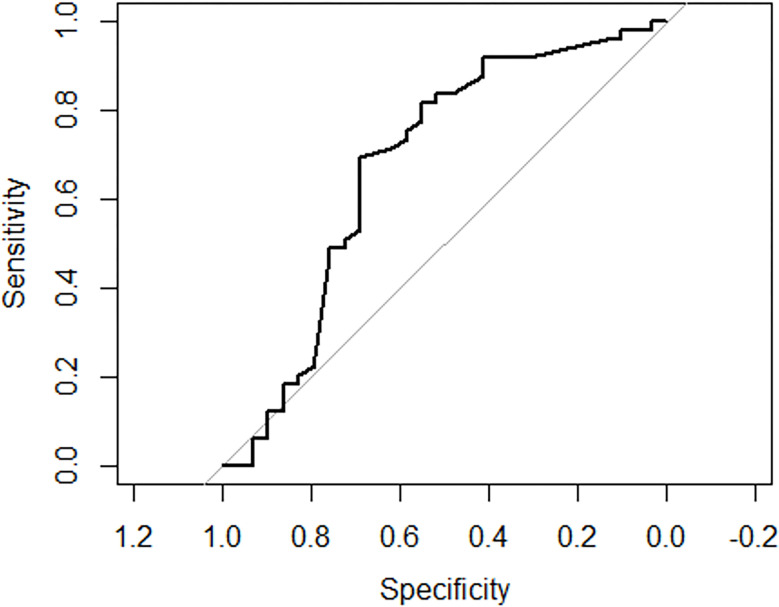
Receiver operating characteristic (ROC) curves for intraoperative complications occurrence following neurosurgical evacuation among adult TBI with EIH Percentage of zeros: 0.9813665 Area under the curve: 0.6752

Holding the rest of variables constant, undergoing fronto-parieto-temporal decompression would make the odds of getting intraoperative complications become 13.329105 times (p = 0.00027). Holding the rest of variables constant, undergoing parieto-temporal decompression would make the odds of getting intraoperative complications become 2.3774298 times (p = 0.04745). Holding the rest of variables constant, undergoing occipito-parietal decompression would make the odds of getting intraoperative complications become 5.2172998 times (p = 0.06236) (Table 4) and (Fig. 4).

### Models to predict mortality after neurosurgical evacuation among traumatic expansive intracranial hematoma patients

**Figure 5:**
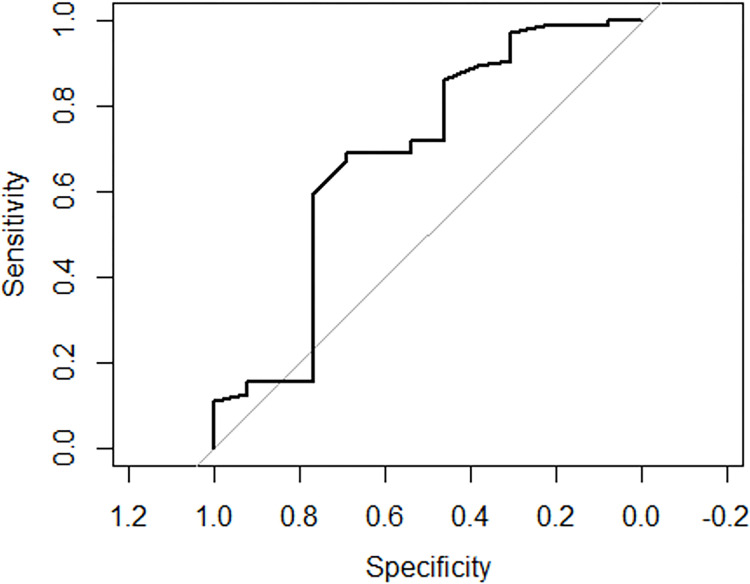
Receiver operating characteristic (ROC) curves for mortality after neurosurgical evacuation among traumatic expansive intracranial hematoma patients Percentage of zeros: 0.9076433 Area under the curve: 0.3667

Holding the rest of variables constant, undergoing craniotomy would make the odds of dying become 10.094291 times (p = 0.000623). Holding the rest of variables constant, undergoing craniotomy would make the odds of dying become 10.094291 times (p = 0.000623). Holding the rest of variables constant, undergoing decompressive craniectomy would make the odds of dying become 1.9833354 times (p = 0.421221) (Table 5) and (Fig. 5).

### Models to predict unfavorable quality of life after neurosurgical evacuation among traumatic expansive intracranial hematoma patients

**Figure 6:**
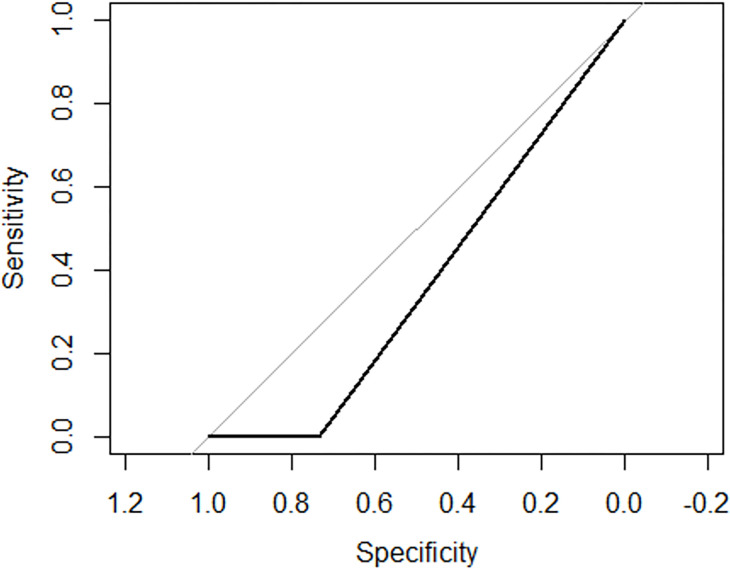
Receiver operating characteristic (ROC) curves for mortality after neurosurgical evacuation among traumatic expansive intracranial hematoma patients Percentage of zeros: 0.9965636 Area under the curve: 0.6164

Health related quality of life of intracranial hematomas after TBI who undergoing surgical evacuation increases overtime. Health-related quality of life of intracranial hematomas after TBI, assessed 6 months post-surgical evacuation, was higher for patients with no EIH (Quality of Life after Brain Injury score: 88.0(8.6) vs 85.0(9.5), P = 0.010) (Table 6) and (Fig. 6).

**Figure 7:**
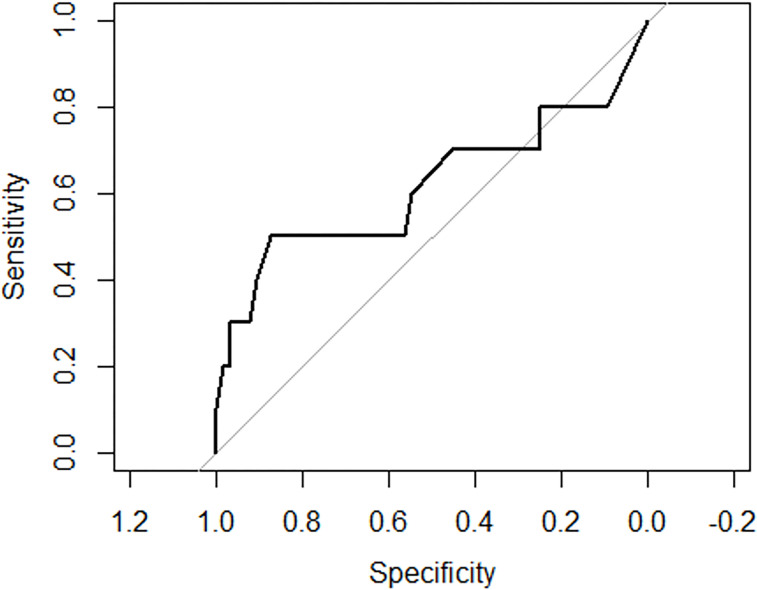
Receiver operating characteristic (ROC) curves for unfavorable quality of life after neurosurgical evacuation among traumatic expansive intracranial hematoma patients Percentage of zeros: 0.9891304

Holding the rest of variables constant, undergoing craniotomy would make the odds of having unfavorable quality of life become 94.632408 times (p = 0.000642). Holding the rest of variables constant, undergoing decompressive craniectomy would make the odds of having unfavorable quality of life become 2.2630195 times (p = 0.639813) (Table 7) and (Fig. 7).

### Models to predict unfavorable functional outcomes after neurosurgical evacuation in traumatic expansive intracranial hematoma patients

Holding the rest of variables constant, undergoing decompressive craniectomy would make the odds of having unfavorable functional outcomes become 2.2412587 times (p = 0.023181). Holding the rest of variables constant, undergoing craniotomy would make the odds of having unfavorable functional outcome become 1.0187434 times (p = 0.000824) (Table 8).

## Discussion

This study assessed the burden, identify relevant risk factors, the interactions between variables, and establish a predictive model for EIH occurrence and surgical evacuation outcomes among TBI patients at Mulago National Referral Hospital. The application of these models can assist in decision-making for individual TBI patients with risk of EIH. Several predictive models for intracranial hematomas have been described by regulatory bodies, most of which focus on the quality of hospitalization ^32^. The evolutionary character of cerebral bleeding and effect of timing from injury (as observed in practice and time of presentation at the unit) to cranial surgery on outcomes are not taken into account by the majority of models ^33^. Furthermore, the models were established on small samples, many were methodologically flawed, and few were validated in external populations ^33^. Few others were neither presented in a clinically practical way, nor were they established in populations in LMIC, where 93% of TBI occur ^34^. Furthermore, patients and surgeons frequently are faced with difficult decisions making in regard to the management of EIH. Weighing in patient characteristics when several options (e.g., age, history of toxic substances, heathy ASA state, imaging findings, evolution of hematoma over time, blood pressure parameters, type of surgery, conservative, golden hour, site of surgery) are available is currently done in an arbitrary way. The development of EIH-specific models would be the cornerstone of defining quality in surgical health care delivery^32^.

### Models that can predict expansive intracranial hematomas occurrence

Considering the feature importance in our developed random forest algorithm-based model, the top five features were age, systolic blood pressure, diastolic blood pressure, subdural hematoma (SDH), diffuse axonal injury (DAI), skull fracture, and an interaction between skull fracture and SDH and were found to be indepently associated with EIH in the final model (Table 2). These findings concur with previous studies which reported that SDH, skull fracture are risk factors to EIH ^12,23–26^. Some studies have linked the evolution of intracranial hematomas with factors such as age greater than 61 ^23,24,35–37^ and elevated admission systolic blood pressure ^23,38,39^. This study did not find subarachnoid hemorrage(SAH) among risk factors as reported by Allison et al.^24^. The average area under the receiver curve (AUC) from a five-fold cross-validation was 0.833, while the average accuracy was 73.4%. The AUC of the current study seven-point models disagree with models developed by Cepata et al (0.72) ^40^, but higher than a simple four-point predictive score reported by Allison et al.(0.77) ^24^.

### Models that can predict intraoperative complications of EIH in TBI patients

Undergoing craniectomy, having a delayed surgery over 72 hours, undergoing frontoparietal, frontoparietotemporal and parietotemporal decompression were found to be independently associated with intraoperative complications in the final model (Table 4). Holding the rest of variables constant, undergoing craniotomy would make the odds of getting intraoperative complications become 1.5244901 times (p = 0.562) while undergoing decompressive craniectomy would make the odds of getting intraoperative complications become 8.5317969 times (p = 0.03578). The fact that patients run the danger of a sizable number of complications after DC and that it may further degrade quality of life is undeniable, even if one were to argue that the technique only reduces mortality at the expense of increasing the proportion of the seriously crippled^41^. The Monro Kellie doctrine’s space restrictions are intended to be circumvented through decompressive craniectomy (DC), which disturbs the cerebral blood and CSF flow dynamics. The timing of the resulting difficulties, which occur days to months following the operation, can be predicted to help in managing them^41^. This finding is in line with a study conducted by Hawryluk et al which confirmed that DC remains a controversy ^30^. Choosing to perform a DC is still challenging and the overall benefits should be balanced against the outcomes and complications on a case by-case basis. DC causes serious complications including meningitis, subdural hygroma, hydrocephalus and increased reoperation rate. Morbidity related to the surgical evacuation was also examined. Intraoperative complications occurred in 58.0% of our patients; the high intraoperative complications confirmed substantial challenges in surgical intervention in LMIC which may impact on the long-term survival and outcomes. Early posttraumatic seizures (PTS) were the most common complication and was observed in 15.7% of patients. PTS are a serious consequence among TBI patients with EIH globally and most especially in low developed countries like Uganda. PTS are commonly associated with severe TBI and the reported incidences vary greatly^42^. In the present study, coma and brain oedema were observed in 11.7% among patients. Furthermore, wound infection (4.9%) and pneumonia (3.4%), CSF leakage (0.3%) were reported in the analysis of 324 patients (Table 4). These results differ from a case series conducted in India where infection (9.1%) and CSF leakage (9.1%) were most reported ^43^.

### Models to predict intraoperative mortality after neurosurgical evacuation among traumatic expansive intracranial hematoma patients

The overall mortality rate in this study was 10.2%, which is lower than the 20% overall mortality rate seen in a prospective research in China on patients who underwent early versus late craniectomy after a traumatic brain injury ^44^. The mortality rate observed in this study agrees with the findings obtained from a study conducted in Uganda (9.6%)^6^. Undergoing craniotomy was found to be independently associated with intraoperative mortality in the final model (Table 5). Holding the rest of variables constant, undergoing craniotomy would make the odds of dying become 10.094291 times (p = 0.000623) while undergoing decompressive craniectomy would make the odds of dying become 1.9833354 times (p = 0.421221) (Table 7). Even if DC was associated with high odds of getting intraoperative complications, however, it was also linked with reduced mortality in this study. These findings were supported by DECRA and RESCUEicp results which reported that the reduced mortality rate and higher rates of complication as a result of DC ^45–47^. This procedure has been demonstrated to reduce ICP and to minimize days in the ICU. A large frontotemporoparietal DC (not less than 12 × 15 cm or 15 cm diameter) is recommended over a small frontotemporoparietal DC for reduced mortality and improved neurologic outcomes in patients with severe TBI^48,49^.

### Models to predict 18 months unfavorable quality of life after neurosurgical evacuation among traumatic expansive intracranial hematoma patients

Undergoing craniotomy and having delayed EIH progression were found to be independently associated with 18 months unfavorable quality of life after neurosurgical evacuation in the final model (Table 7). Holding the rest of variables constant, undergoing craniotomy would make the odds of having unfavorable quality of life become 94.632408 times (p = 0.000642) while undergoing decompressive craniectomy would make the odds of having unfavorable quality of life become 2.2630195 times (p = 0.639813). (Table 7). Health related quality of life of intracranial hematomas after TBI who undergoing surgical evacuation increased overtime. Health-related quality of life of intracranial hematomas after TBI, assessed 6 months post-surgical evacuation, was higher for patients without EIH (Quality of Life after Brain Injury score: 88.0(8.6) vs 85.0(9.5), P = 0.010) (Table 7). This finding agrees with previous study which demonstrated that EIH are the most dangerous form of intracranial hematoma that occurs following traumatic brain injury (TBI)^38,50^. In addition, EIH appears to be associated with a high rate of neurological deterioration in patients with TBI^20,21^. What is not contested is that EIH patients face the risk of a large number of complications after the operation and that can further compromise their quality of life.

### Models to predict unfavorable functional outcomes after neurosurgical evacuation in traumatic expansive intracranial hematoma patients

However, 88.4% (282) patients survived from intracranial hematoma following TBI. Among these patients, 73.8% (208) were discharged with favourable condition (defined by GOS of > 3) while 26.2% (74) were discharged with unfavourable condition (defined by GOS of < 3). These findings are better when compared with a study conducted at MNRH which reported 71.7% patients were alive at the end of the study ^51^. Undergoing craniotomy, craniectomy and having delayed EIH progression were found to be independently associated with 18 months unfavorable functional outcomes after neurosurgical evacuation in the final model (Table 8). Holding the rest of variables constant, undergoing decompressive craniectomy would make the odds of having unfavorable functional outcomes become 2.2412587 times (p = 0.023181) while undergoing craniotomy would make the odds of having unfavorable functional outcome become 1.0187434 times (p = 0.000824). (Table 8). This result is in line with previous reports where DC has been established to reduce mortality only at the expense of increasing the proportion of the severely disabled ^49^.

The present study had limitations including, small sample size, more variables of 824 than the sample size of the study participants and missing values. This was expected and the research team increased 10% of possible dropout or missing data. Thus, the model selection was done to achieve better interpretability of the findings. The model used a 70% and 30% split for the train and test data. A seed was set so that the findings were reproducible. However, the findings show that the selected models were subject to changes when the seed changes, meaning a person who uses a different configuration for the 70%, 30% is likely to get different models in the positive direction. And these findings could be due to the small sample size used in the study. In addition, there were not enough samples to fit a large model with all these factors and their interaction and then do model selection.

## Conclusion

We developed 5 proposed models including (1) model with 8 coefficients for EIH occurrence; (2) model for intraoperative complications; (3) model for death occurrence; (4) model for unfavorable quality of life and (5) model for unfavorable functional outcomes which have 15 coefficients each to predict earlier individualized estimates of EIH occurrence and surgical evacuation outcomes based on preoperative conditions at MNRH and similar settings across the region. These proposed models have demonstrated good discrimination with AUC for EIH occurrence (0.833) while the average accuracy was 73.4%, intraoperative complications (0.67), mortality (0.36), and 18 months unfavorable quality of life after neurosurgical evacuation in TBI patients (0.61).

## Figures and Tables

**Table 1:** Baseline demographic characteristics of patients with EIH

Variables	No expansive hematoma No. (%)	Expansive hematoma No. (%)	Total No. (%)	P-Value
n (%)	132 (40.7)	192 (59.3)	324 (100.0)	
Age in complete years, mean (sd)	30.5 (14.0)	42.3 (17.9)	37.5 (17.4)	<0.001
**Gender, n (%)**				
Female	24 (18.2)	39 (20.3)	63 (19.4)	
Male	108 (81.8)	153 (79.7)	261 (80.6)	0.634
**Patients’ occupation, n (%)**				
Farming	11 (8.3)	26 (13.5)	37 (11.4)	
Family business	16 (12.1)	38 (19.8)	54 (16.7)	
Government worker	6 (4.5)	8 (4.2)	14 (4.3)	
Industry worker	3 (2.3)	5 (2.6)	8 (2.5)	
Boda rider	72 (54.5)	80 (41.7)	152 (46.9)	
Taxi driver	23 (17.4)	29 (15.1)	52 (16.0)	
Other, specify	1 (0.8)	6 (3.1)	7 (2.2)	0.136
**Residence type / location, n (%)**				
Rural	78 (59.1)	106 (55.2)	184 (56.8)	
Urban	54 (40.9)	86 (44.8)	140 (43.2)	0.488
**Region, n (%)**				
Central	95 (72.0)	135 (70.3)	230 (71.0)	
Eastern	10 (7.6)	9 (4.7)	19 (5.9)	
Northern	8 (6.1)	27 (14.1)	35 (10.8)	
Western	19 (14.4)	21 (10.9)	40 (12.3)	0.088
**Matrimonial state, n (%)**				
Unmarried	62 (47.0)	65 (33.9)	127 (39.2)	
Married	70 (53.0)	127 (66.1)	197 (60.8)	0.017

**Table 2:** Model coefficients for expansive intracranial hematomas including demographic, blood pressure, imaging characteristic of patients with traumatic brain injury and their significance level

	Odds Ratio	Pr(>|z|)
(Intercept)	0.1432344	0.1931110
Age 18 to 30	0.4644902	0.1493704
Age 31 to 45	0.9938626	0.9918531
Systolic blood pressure	1.0451607	0.0062050*
Diastolic pressure	0.9419734	0.0069345*
Skull fracture	1.8982746	0.2802460
Subdural hematoma	6.2860509	0.0038340*
Diffuse axonal injury	4.0244741	0.0081517*
Skull fracture and subdural hematoma	0.0675804	0.0026879*

Key codes : 0 ‘***’ 0.001 ‘**’ 0.01 ‘*’ 0.05 ‘.’ 0.1 ‘ ’ 1 — Statistical significance at 95% CI

**Table 3:** Baseline complications occurrence for adult traumatic brain injury patients with intracranial hematomas

Complications, n (%)	No EIH 132 (40.7)	EIH 192 (59.3)	Total 324 (100.0)	p-Value
No	53 (40.2)	83 (43.2)	136 (42.0)	
Yes	79 (59.8)	109 (56.8)	188 (58.0)	0.581
Infection (Y), n (%)	8 (6.1)	8 (4.2)	16 (4.9)	0.439
Pneumonia (infection of the lungs) (Y), n (%)	8 (6.1)	3 (1.6)	11 (3.4)	0.028
Early PTS (Y), n (%)	22 (16.7)	29 (15.1)	51 (15.7)	0.704
Brain swelling (Y), n (%)	11 (8.3)	27 (14.1)	38 (11.7)	0.115
Leakage of cerebrospinal fluid) (Y), n (%)	4 (3.0)	5 (2.6)	9 (2.8)	0.816
Subdural hygroma	3(2.3)	1(0.5)	4(1.2)	0.161
Nutrition deficit	5(3.8)	8(4.2)	13(4.0)	0.864
Coma (Y), n (%)	13 (9.8)	25 (13.0)	38 (11.7)	0.383
Death	16 (12.1)	17 (8.9)	33 (10.2)	
Alive	116 (87.9)	175(91.1)	282(88.4)	0.073

**Table 4:** Model coefficients for intraoperative complications occurrence following neurosurgical evacuation among adult TBI with EIH

	Odds Ratio	Pr(>|z|)
(Intercept)	4.183465	0.13097
Craniotomy	1.5244901	0.56229
Craniectomy	8.5317969	0.03578 *
Late EIH evolution	1.7697884	0.20932
Delayed EIH evolution	1.0411334	0.92964
Early surgery < 24 hours	1.6229086	0.27600
Delay surgery > 72 hours	2.8757974	0.00229 **
Frontoparietal	3.6784446	0.00531 **
Fronto-parieto-temporal	13.329105	0.00027 ***
Parieto-temporal	2.3774298	0.04745 *
Occipito-parietal	5.2172998	0.06236.
Temporal	1.2322478	0.70056
Parietal	2.2086688	0.15887
Occipital	1.1721855	0.99435

Key codes : 0 ‘***’ 0.001 ‘**’ 0.01 ‘*’ 0.05 ‘.’ 0.1 ‘ ’ 1 — Statistical significance at 95% CI

**Table 5:** Model coefficients for death occurrence following neurosurgical evacuation among adult TBI with EIH

	Odds Ratio	Pr(>|z|)
(Intercept)	1.2800511	0.815856
Craniotomy	10.094291	0.000623 ***
Craniectomy	1.9833354	0.421221
Late EIH evolution	1.1220529	0.870493
Delayed EIH evolution	1.8730161	0.356094
Early surgery < 24 hours	1.4358399	0.589988
Delay surgery > 72 hours	2.0538785	0.166506
Frontoparietal	2.4126844	0.251787
Fronto-parieto-temporal	2.9897805	0.069283
Parieto-temporal	1.2655793	0.730060
Occipito-parietal	7621090.2	0.989277.
Temporal	1.6203626	0.540545
Parietal	5.2744794	0.140446
Occipital	3591174.9	0.996956
Interhemispheric	9.752756	0.142329
Posterior fossa	4531483.8	0.995597

Key codes : 0 ‘***’ 0.001 ‘**’ 0.01 ‘*’ 0.05 ‘.’ 0.1 ‘ ’ 1 — Statistical significance at 95% CI

**Table 6:** Baseline quality of life patterns

Quality of life after surgical evacuation in TBI pateints	No expansive hematoma No. (%)	Expansive hematoma No. (%)	Total No. (%)	P-Value
n (%)	132 (40.7)	192 (59.3)	324 (100.0)	
Overall QOL first 24 hrs, mean (sd)	50.0 (15.3)	49.0 (16.8)	49.4 (16.2)	0.596
Overall QOL 30 days, mean (sd)	61.6 (19.6)	62.2 (17.2)	61.9 (18.2)	0.771
Overall QOL 90days, mean (sd)	79.4 (9.5)	76.3 (10.4)	77.5 (10.1)	0.011
Overall QOL 180 days, mean (sd)	88.0 (8.6)	85.0 (9.5)	86.0 (9.3)	0.010

**Table 7:** Model coefficients for unfavorable quality of life following neurosurgical evacuation among adult TBI with EIH

	Odds Ratio	Pr(>|z|)
(Intercept)	32859.625	8.3e-09 ***
Craniotomy	94.632408	0.000642 ***
Craniectomy	2.2630195	0.639813
Late EIH evolution	1.1220529	0.31431
Delayed EIH evolution	1.8730161	0.03095 *
Early surgery < 24 hours	2.49328221	0.730551
Delay surgery > 72 hours	2.0293184	0.889183
Frontoparietal	1.3899948	0.741435
Fronto-parieto-temporal	2.9897805	0.069283
Parieto-temporal	2.5602374	0.670343
Occipito-parietal	1.1917458	0.914285.
Temporal	1.4207714	0.757920
Parietal	2.4084900	0.434468
Occipital	60.448998	0.397900
Interhemispheric	1.3996189	0.923242
Posterior fossa	9.3352728	0.521610

Key codes : 0 ‘***’ 0.001 ‘**’ 0.01 ‘*’ 0.05 ‘.’ 0.1 ‘ ’ 1 — Statistical significance at 95% CI

**Table 8:** Model coefficients for unfavorable functional outcomes after neurosurgical evacuation in adult TBI with EIH

	Odds Ratio	Pr(>|z|)
(Intercept)	5.8789607	5.3e-09 ***
Craniotomy	1.0187434	0.000824 ***
Craniectomy	2.2412587	0.023181*
Late EIH evolution	2.3471276	0.11431
Delayed EIH evolution	0.4913294	0.02595 *
Early surgery < 24 hours	0.4645543	0.530551
Delay surgery > 72 hours	1.6711724	0.389183
Frontoparietal	1.0032362	0.341435
Fronto-parieto-temporal	3.3292700	0.059283
Parieto-temporal	0.1039742	0.680343
Occipito-parietal	0.3675388	0.114285.
Temporal	1.8972791	0.257920
Parietal	3.5065020	0.334468
Occipital	7.4408072	0.197900
Interhemispheric	7.1218168	0.923242
Posterior fossa	2.1719616	0.521610

Key codes : 0 ‘***’ 0.001 ‘**’ 0.01 ‘*’ 0.05 ‘.’ 0.1 ‘ ’ 1 — Statistical significance at 95% CI

## Data Availability

Datasets used in the current study are available from the corresponding author upon reasonable request.

## List Of Abbreviations

EIH: Expansive intracranial hematoma
GCS: Glasgow coma scale
GOS: Glasgow outcome scale
HIC: High-income countries
LMIC: Low-and middle-income countries
MDS1: Midline shift <1cm
MDS: Midline shift ≥1 cm
MNRH: Mulago National Referral Hospital
OBC: Open basal cistern
PR: Prevalence risk
QoLIBRI: quality of life after brain Injury
RCT: randomized clinical trials
TBI: Traumatic Brain Injury
TEH: Traumatic expansive hematoma
SAH: Subarachnoid hemorrhage
SDH: Subdural hematoma
SK: Skull fracture
MAP: Mean arterial pressure
